# Interleukin-12 inhibits pathological neovascularization in mouse model of oxygen-induced retinopathy

**DOI:** 10.1038/srep28140

**Published:** 2016-06-17

**Authors:** Yedi Zhou, Shigeo Yoshida, Yuki Kubo, Yoshiyuki Kobayashi, Takahito Nakama, Muneo Yamaguchi, Keijiro Ishikawa, Shintaro Nakao, Yasuhiro Ikeda, Tatsuro Ishibashi, Koh-Hei Sonoda

**Affiliations:** 1 Department of Ophthalmology, Kyushu University Graduate School of Medical Sciences, Fukuoka, 812-8582, Japan

## Abstract

Hypoxia-induced retinal neovascularization is a major pathological condition in many vision-threatening diseases. In the present study, we determined whether interleukin (IL)-12, a cytokine that regulates angiogenesis, plays a role in the neovascularization in a mouse model of oxygen-induced retinopathy (OIR). We found that the expressions of the mRNAs of both IL-12p35 and IL-12p40 were significantly reduced in the OIR retinas compared to that of the room air-raised control. The sizes of the avascular areas and neovascular tufts were larger in IL-12p40 knock-out (KO) mice than that in wild type (WT) mice. In addition, an intravitreal injection of recombinant IL-12 reduced both avascular areas and neovascular tufts. IL-12 injection enhanced the expressions of interferon-gamma (IFN-γ) and other downstream chemokines. In an *in vitro* system, IL-12 had no significant effect on tube formation of human retinal microvascular endothelial cells (HRECs). Moreover, a blockade of IFN-γ suppressed the inhibitory effect of IL-12 on pathological neovascularization. These results suggest that IL-12 plays important roles in inhibiting pathological retinal neovascularization.

Hypoxia is a leading mechanism that induced angiogenesis, and retinal neovascularization is a major pathological condition in many vision-threatening diseases including proliferative diabetic retinopathy, retinal vein occlusion and retinopathy of prematurity^1^. These proliferative vascular diseases may lead to irreversible damage to the patients. Oxygen-induced retinopathy (OIR), a mouse model for retinopathy of prematurity and other hypoxia-induced retinal diseases, is widely used for the studies of retinal neovascularization^2^. In this model, two types of retinal neovascularization exist: pathological neovascularization and physiological revascularization. The previous one occurs with the sprouting of abnormal vessels that grow from the inner layer of the retina into the vitreous, and the other is against avascular areas with functional intraretinal vessels^2^,^3^.

We reported that the M2 macrophages, rather than the M1 macrophages, played essential roles in enhancing pathological retinal neovascularization in OIR model, and the majority of those macrophages had the M2 phenotype^3^. M1 macrophages express high levels of interleukin (IL)-12, IL-18, and IL-23, and low levels of IL-10^4^,^5^. In contrast, M2 macrophages produce high levels of IL-10 and low levels of IL-12 and IL-23^6^,^7^. This difference is significant as it has been reported that IL-10 promotes pathological neovascularization^4^, while IL-18 regulates pathological neovascularization negatively through the regression of blood vessels in the OIR model^8^.

IL-12 is an important cytokine that is naturally produced by antigen-presenting cells (APCs) including dendritic cells, monocytes, macrophages, and B cells after toll-like receptor engagement^9^,^10^. IL-12 is a heterodimeric cytokine encoded by two genes, p35 and p40 chain subunits, and these can combine to the active p70 heterodimer^11^,^12^,^13^. IL-12 induces the synthesis of IL-10, but IL-10 suppresses IL-12 production. This interaction is regarded as a negative feedback mechanism^14^,^15^,^16^. IL-12 is also a key factor of the immune responses of Th1 cells by inducing the production of IFN-γ which is considered to be a key cytokine in the Th1 response^17^,^18^,^19^. It has also been reported that CD4+ and CD8+ T cells are involved in the activation of IL-12^20^,^21^. On the other hand, IL-12 exposure leads to the infiltration of CD4+ and CD8+ T cells in antitumor studies^22^.

It is known that IL-12 inhibits angiogenesis in several systems including corneal neovascularization and tumor angiogenesis^10^,^23^,^24^,^25^,^26^,^27^,^28^. The concentrations of IL-12 in the aqueous humor is significantly higher in patients with neovascularization associated with diabetic retinopathy^29^, and higher concentrations of IFN-γ were detected in the vitreous of the patients with diabetic retinopathy^30^. Macrophages in IL-12p40-deficient mice have a bias to M2 activation from M1 phenotype^31^. In spite of all of these findings, the role of IL-12 in retinal neovascularization has not been fully understood.

From what we discussed above, we hypothesized that IL-12 might be involved in retinal neovascularization and could also be a potential therapeutic target. In the present study, we investigated the role played by IL-12 in retinal neovascularization of the OIR mouse model. The possible mechanism(s) of the IL-12 in retinal neovascularization were also presented.

## Results

### Down-regulation of mRNA of IL-12 after retinal ischemia

**Figure 1:**
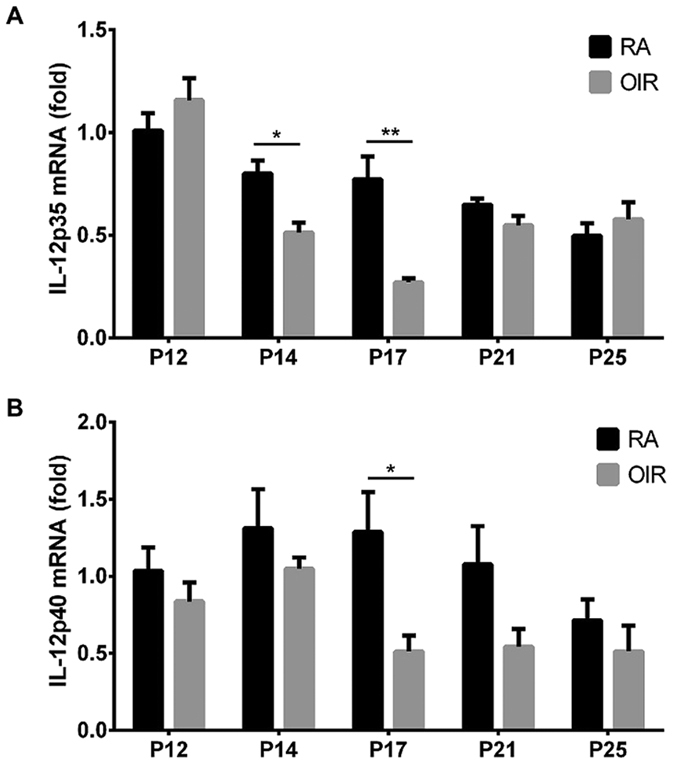
Expression of the mRNAs of IL-12p35 and IL-12p40 determined by real-time RT-PCR in retinas with oxygen-induced retinopathy (OIR). (**A**) Expression of IL-12p35 was significantly decreased at P14 and P17. (**B**) Expression of IL-12p40 was significantly decreased at P17. ^**^P < 0.01, ^*^P < 0.05 compared to the room air controls at the same time points (n = 4/group).

To investigate the expression of IL-12 during the development of retinal neovascularization, we determined the expressions of the mRNAs of IL-12p35 and IL-12p40 in the retinas of the OIR mouse model. Total mRNA was extracted from whole retinas of C57BL/6J mice pups with or without OIR at several selected time points. The results of real-time PCR showed that mRNA expression of IL-12p35 was significantly decreased at P14 and P17 compared to the control retinas (Fig. 1A). In addition, the expression of IL-12p40 was also significantly reduced at P17 (Fig. 1B). Similar to IL-12, the expression of IFN-γ was significantly decreased at P17 (Supplementary Fig. S1). These data indicate that the expression of the mRMA of IL-12 was reduced during pathological neovascularization.

### Deficiency of IL-12 enhances retinal neovascularization

**Figure 2:**
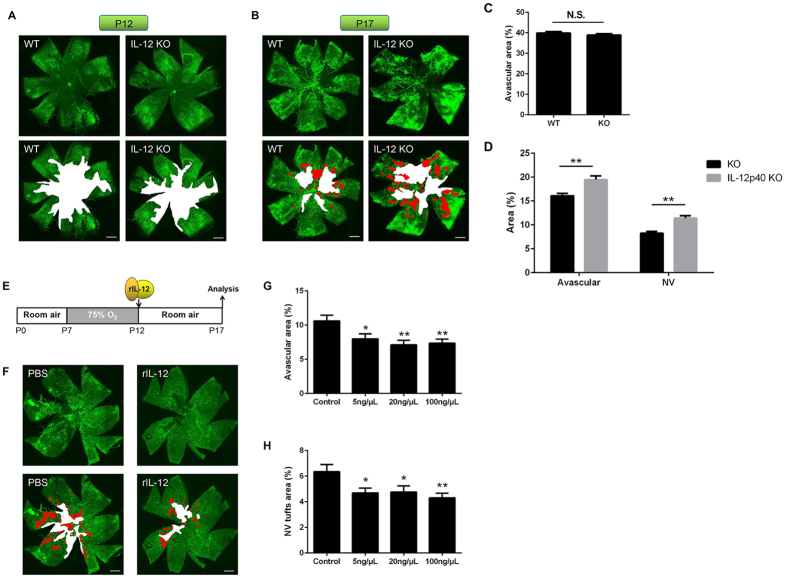
Suppressive effect of neovascularization by IL-12 in OIR retinas. (**A**) Representative images of isolectin B4-stained retinal flat-mounts at P12 of WT (left) and IL-12p40 KO mice (right). (**B**) Representative images of isolectin B4-stained retinal flat-mounts at P17 of WT (left) and IL-12p40 KO mice (right). (**C**)Quantitative assessment of the avascular areas at P12 of WT and IL-12p40 KO mice (n = 8/group). (**D**) Quantitative assessment of avascular areas and neovascular tufts areas at P17 of WT and IL-12p40 KO mice (n = 8/group). Both avascular areas and neovascular tufts were more evident in the IL-12p40 KO mice than the WT control at P17. (**E**) Time course of the experiments with IL-12 injection. rIL-12 or PBS was injected into the vitreous at P12 immediately after returning the mice to room air. The eyes were analyzed at P17. (**F**) Representative images of isolectin B4-stained retinal flat-mounts at P17 in mice that were injected with PBS (left) and rIL-12 (right). (**G**) Quantitative assessment of avascular areas in the mice that were injected with PBS and rIL-12 (n = 12/group). (**H**) Quantitative assessment of neovascular tufts areas in the mice that were injected with PBS and rIL-12 (n = 12/group). Both avascular areas and neovascular tufts were smaller in the mice injected with rIL-12 than with PBS as control at P17. ^**^P < 0.01, ^*^P < 0.05 compared to the controls. *Scale bars:* 500 μm.

To determine if IL-12 affects retinal neovascularization, wild type (WT) and IL-12p40 knock-out (KO) mice were exposed to hyperoxygen from P7 to P12, and then returned to the room air. The retinal avascular areas (white color) in the IL-12p40 KO mice (38.91 ± 0.69%) were not significantly different from the WT (39.91 ± 0.76%) control at P12 (*P* > 0.05; n = 8, Fig. 2A,C). However, at P17 when pathological neovascularization was at its maximum, the IL-12p40 KO mice had significant larger pathological neovascular tufts (red color; 11.42 ± 0.48% vs 8.23 ± 0.38%; *P* < 0.01, n = 8) and avascular areas (white color; 19.48 ± 0.78% vs 16.10 ± 0.50%; *P* < 0.01, n = 8, Fig. 2B,D). Therefore, deficiency of IL-12p40 enhanced the pathological neovascularization in mice with OIR.

### IL-12 regulation of retinal neovascularization

To confirm that IL-12 plays a role in retinal neovascularization, we injected recombinant IL-12 (rIL-12) into the vitreous immediately after returning the pups to room air on P12. Examination of the retinas collected on P17 showed that the avascular areas (white color) were reduced in the mice treated with rIL-12 at concentrations of 5 ng/μL (7.98 ± 0.74%), 20 ng/μL (7.12 ± 0.65%), and 100 ng/μL (7.36 ± 0.59%) compared to the control group (10.61 ± 0.83%; *P* < 0.05 and *P* < 0.01, n = 12/group, Fig. 2F,G).

The size of the pathological neovascular tufts (red color) were also reduced in the mice treated with rIL-12 at a concentration of 5 ng/μL (4.70 ± 0.37%), 20 ng/μL (4.76 ± 0.48%), and 100 ng/μL (4.30 ± 0.37%) compared to the control group (6.35 ± 0.56%; *P* < 0.05 and *P* < 0.01, n = 12/group, Fig. 2F,H).

These results indicate that IL-12 had anti-angiogenic effects on pathological neovascularization and promoted physiological revascularization.

### Effects of IL-12 on expression of cytokines and growth factors in retinas of OIR mice

**Figure 3:**
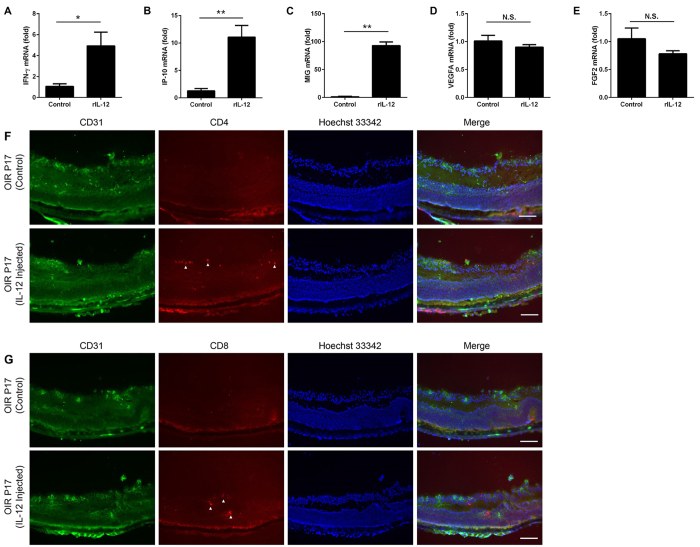
Effect of intravitreal IL-12 on the expressions of cytokines and the infiltration of CD4+ and CD8+ T cells in OIR retinas. Expressions of IFN-γ (**A**), IP-10 (**B**), and MIG (**C**) were significantly increased in the eyes that were injected with rIL-12. However, the expressions of VEGFA (**D**) and FGF2 (**E**) are not significantly changed in the rIL-12 injected group. Double staining for CD31 (green) and CD4 or CD8 (red) in cryostat sections of OIR eyes that were injected with PBS or rIL-12. Some CD4+ (**F**) and CD8+ cells (**G**) infiltrated in the retina (arrowheads) of the rIL-12 injected group. ^**^P < 0.01, ^*^P < 0.05 compared to the controls that were injected with PBS (n = 4/group). *Scale bars*: 100 μm.

To further investigate the mechanisms that caused by IL-12 on retinal neovascularization, we injected rIL-12 or PBS intravitreally in pups at day 12 when they were returned to room air. We determined the level of expression of the downstream cytokines and growth factors in the whole retinas at P17. After the intravitreal injection of rIL-12, the mRNA expressions of IFN-γ, monokine-induced by gamma interferon (CXCL9/MIG), and interferon-inducible protein 10 (CXCL10/IP-10) were markedly increased compared to that of the control group (*P* < 0.05, *P* < 0.01, n = 4; Fig. 3A–C). The expressions of VEGFA and FGF2 were not significantly reduced after injection of rIL-12 (*P* = 0.341 and 0.223 respectively, *n* = 4, Fig. 3D,E). These findings indicate that IL-12 stimulated the synthesis of its downstream cytokines which probably are the factors inhibiting the neovascularization in the OIR retinas.

### Infiltration of T cells in IL-12 treated retinas

To determine whether CD4− and CD8− positive T cells had infiltrated the retina of IL-12-injected mice, we injected rIL-12 into the vitreous, and then performed immunofluorescence staining using antibodies for CD4 and CD8 together with the endothelial cell marker CD31. The results showed that CD4+ and CD8+ T cells had infiltrated into the IL-12-treated retinas, and some of the T cells were associated with the neovascular tufts (Fig. 3F,G). In addition, fewer CD4+ and CD8+ T cells were found in the retina of PBS-injected eyes (Fig. 3F,G). These results indicated that IL-12 might stimulate the infiltration of T cells in the OIR retinas.

### No direct effect of IL-12 on tube formation by retinal endothelial cells (HRECs) in culture

**Figure 4:**
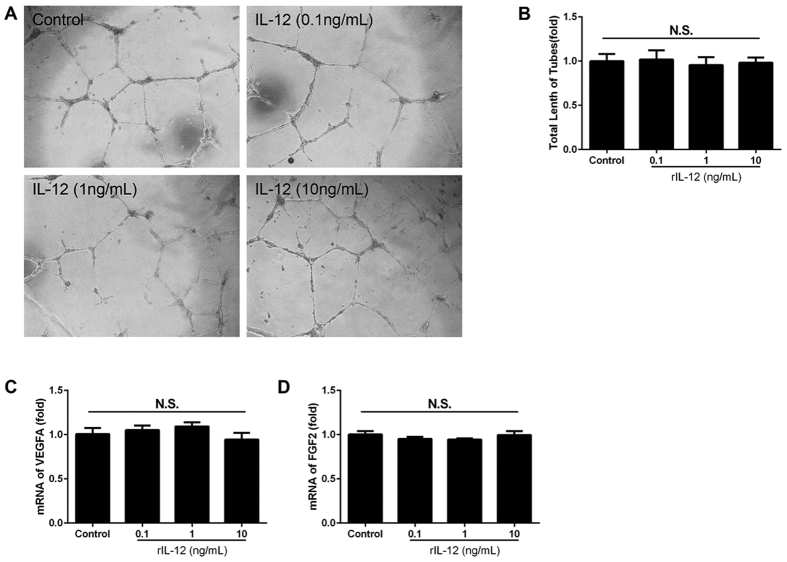
IL-12 has no direct effect on the growth of human microvascular retinal epithelial cells (HRECs) *in vitro*. Photographs of tube formation were taken after cultured alone or stimulated by rIL-12 (**A**). The lengths of the tubes were quantitative assessed in each group. The total lengths were not significantly different among the groups (**B**, n = 12/group). The mRNA expressions of VEGFA (**C**) and FGF2 (**D**) were not changed after exposure to rIL-12 (n = 4/group). *P* > 0.05 compared to the controls without rIL-12.

To examine whether IL-12 has direct effects on HRECs, we cultured HRECs in a tube formation assay with or without human rIL-12. The total length of the tubes in the groups that were cultured with rIL-12 had no significant difference compare to the control group (*P* = 0.960, *n* = 12, Fig. 4A,B). However, both MIG and IP-10 inhibited tube formation by HRECs (Supplementary Fig. S2).

To confirm the effects of IL-12 on the change in the expression of its downstream cytokine IFN-γ and growth factors, we cultured HRECs with or without human rIL-12. Then, the cells were collected and mRNA expressions of cytokines were determined by real-time RT-PCR. The results showed that the expressions of both VEGFA and FGF2 were not affected 24 h after exposure to rIL-12 (*P* = 0.397 and 0.495 respectively, *n* = 4, Fig. 4C,D). In addition, IFN-γ was not detected in all of the groups. These findings indicate that IL-12 has no direct effect on tube formation by HRECs *in vitro*.

### Suppression of IFN-γ blocks the inhibitory effect of IL-12 on pathological neovascularization

**Figure 5:**
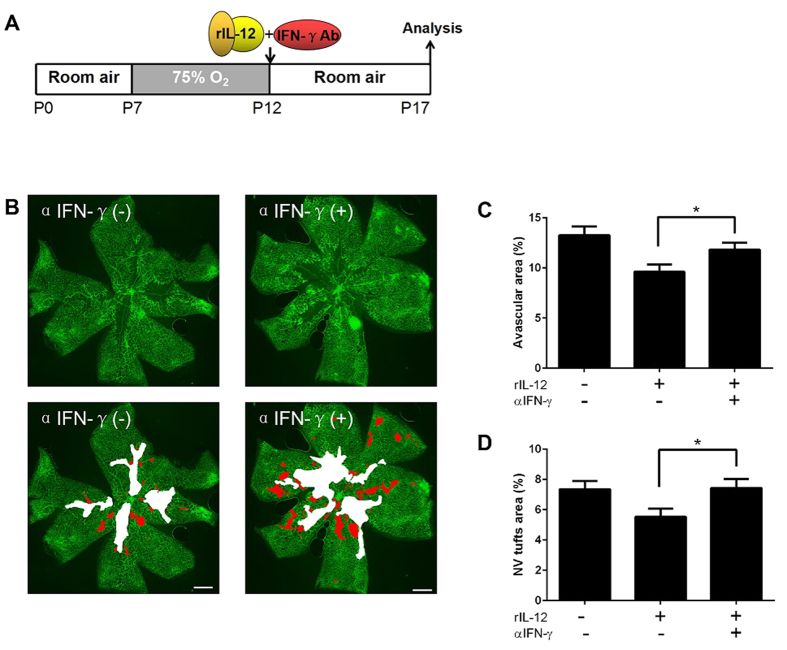
Intravitreal injection of anti-IFN-γ rescues the anti-angiogenic activity of IL-12. (**A**) Time course of the experiments. rIL-12 mixed with anti-IFN-γ antibody or non-immune IgG was injected into the vitreous at P12. The eyes were analyzed at P17. (**B**) Representative images of flat-mounted retinas in the OIR mice that were injected of rIL-12 with non-immune IgG or anti-IFN-γ antibody at P12. (**C**) The size of the avascular areas was significantly larger than that of the controls. (**D**) The size of neovascular tufts was significantly larger than that of the controls. ^*^P < 0.05 compared to the controls injected with rIL-12 and non-immune IgG (n = 12 or 14/group). *Scale bars*: 500 μm.

To confirm whether IFN-γ mediated the anti-angiogenic effects of IL-12 in the retina, we injected rIL-12 mixed with or without anti-IFN-γ neutralizing antibody into the vitreous at P12. The results showed that the group injected with anti-IFN-γ had larger areas of neovascular tufts (red color) compared to the non-immune IgG control group (7.62 ± 0.63% and 5.53 ± 0.53% respectively; *P* < 0.05, *n* = 12 and 14, Fig. 5B,D). Moreover, the size of the avascular areas (white color) was significantly larger than that of the non-immune IgG control group after the injection of anti-IFN-γ antibody (11.74 ± 0.72% and 9.62 ± 0.71% respectively; *P* < 0.05, *n* = 12 and 14, Fig. 5B,C). These results indicated that an inhibition of IFN-γ blocked both the reduction of pathological neovascularization and the enhanced physiological revascularization that caused by IL-12. Thus, IFN-γ might be a key mediator of the anti-angiogenic effects induced by IL-12 in OIR retinas.

## Discussion

The anti-angiogenic activity of IL-12 is achieved by regulating the formation of new blood vessels. As far as we know, this study is the first to show the role of IL-12 in retinal neovascularization. The expressions of the mRNAs of IL-12p35 and IL-12p40 were significantly decreased in OIR retinas (Fig. 1), which was most likely because IL-12 is mainly produced by M1 activation. In our previous studies, we showed that the activation of M2 rather than M1 macrophages played a more important role in retinal neovascularization^3^. This might be one of the reasons for these findings.

The knockout of IL-12p40 enhanced the pathological neovascularization and interfered with the physiological revascularization at P17, but it had no effects on the size of the avascular areas at P12 (Fig. 2). In this model, IL-12 probably regulates the physiological revascularization and the subsequent pathological neovascularization. In addition, injection of rIL-12 inhibited the pathological neovascularization and promoted physiological revascularization (Fig. 2). However, in the *in vitro* studies using HRECs, IL-12 had neither a direct effect on the tube formation nor on the production of growth factors VEGFA and FGF2 (Fig. 3). These observations demonstrated that no direct anti-angiogenic action of IL-12 on HRECs in culture.

The anti-angiogenetic activity of IL-12 is probably indirect and is mediated by IFN-γ production by several kinds of leukocytes^32^,^33^,^34^,^35^. IL-12 and IFN-γ are strongly related to Th1 differentiation and development^19^,^36^,^37^. IP-10 (CXCL10) and MIG (CXCL9) are CXC chemokines which are mainly induced by IFN-γ and both contribute to the anti-tumor effects of IL-12^27^,^28^. An earlier study showed that IL-12 inhibits angiogenesis through the activation of IP-10^38^. In our study, IL-12 injection enhanced the production of IFN-γ as well as the secretion of cytokines MIG and IP-10 in the OIR retinas (Fig. 3). These results indicate that IFN-γ and/or its induced cytokines might mediate the inhibitory effects of IL-12 on pathological retinal neovascularization. In addition, an injection of IL-12 did not reduce the level of VEGFA and FGF2 significantly. Thus, the anti-angiogenic effects induced by IL-12 might through mechanism(s) independent from VEGFA and FGF2. We also noted that IL-12 did not have a direct effect on the tube formation of HRECs (Fig. 4). Instead, MIG and IP-10 inhibited the growth of tube formation of HRECs (Supplementary Fig. 2). It is possible that leukocytes and/or other cells, rather than HRECs, might be necessary for the activation of IFN-γ and its downstream chemokines MIG and IP-10.

IL-12 stimulates the production of IFN-γ by leukocytes including NK cells and T cells^39^,^40^. We injected rIL-12 into the vitreous at P12 and noted that CD4+ and CD8+ T cells infiltrated the IL-12-treated retinas (Fig. 3). This suggests that the IL-12 may enhanced the differentiation and/or recruitment of T cells which then resulted in a secretion of IFN-γ in the OIR retinas.

The role played by IFN-γ in angiogenesis is controversial. Nagineni *et al.* reported that IFN-γ promotes pathological neovascularization in eyes with AMD by enhancing the secretion of VEGF by the HRPE cells^41^. High expression of IFN-γ in ocular tissues in patients with PDR was reported to be an indirect inducer of angiogenesis through the activation of VEGF^42^. However, an intraocular injection of IFN-γ stimulated macrophages and injection of recombinant IFN-γ significantly reduced pathological choroidal neovascularization^43^,^44^. Consistent with these reports, the results of the present study indicated that the inhibitory effect of IL-12 on pathological neovascularization was mediated by IFN-γ. This was found by demonstrating that anti-IFN-γ exposure could not only block the IL-12-induced suppression of pathological neovascularization, but also block the enhanced physiological revascularization in OIR retinas (Fig. 5). This confirmed that IFN-γ is required for the inhibition of IL-12 in retinal neovascularization. Further studies investigating the mechanisms of IL-12-IFN-γ axis might provide important information on pathological neovascularization.

The IL-12 family of cytokines includes IL-12, IL-23, IL-27, and IL-35^45^,^46^. IL-12 shares p35 with IL-35 and p40 with IL-23^46^. Interestingly, it has been reported that IL-35 and IL-23 promote angiogenesis in tumors which might be involved in the Th17 pathway^47^,^48^. Further studies are required to determine the role of IL-12, IL-23, IL-27, and IL-35 in the formation of retinal neovascularization.

We recently reported that the M2 macrophages and their related cytokines were highly expressed in fibrovascular membranes of the patients with proliferative diabetic retinopathy^49^,^50^. M2 rather than M1 macrophages enhanced the pathological neovascularization in the OIR model^3^. M1 and M2 macrophages promote Th1 and Th2 responses respectively, and they produced cytokines of the Th1 and Th2 responses, e.g., IFN-γ and IL-4, which also play some roles in the regulation of M2 and M1 activities respectively^51^. As M2 macrophages play a critical role in retinal neovascularization in this model^3^, the majority of the macrophages should be M2 macrophages polarized from M1 which may lead to the decrease of IL-12 production in the OIR retinas (Fig. 1). It is known that IFN-γ is an essential cytokine that biases the M1 polarization^52^, and it is reasonable that IL-12 stimulation biases the M1 activation. Above all, IL-12 might regulate and also be regulated by M1-M2 activations of macrophages during its inhibition of pathological neovascularization in OIR retinas. This should be examined in more detail in future investigations.

In conclusion, IL-12 inhibits the pathological retinal neovascularization that is mediated by IFN-γ in the mouse model of OIR. Therefore, IL-12 should be considered as a potential therapeutic agent for inhibiting pathological neovascularization.

## Methods

### Mouse Model of oxygen-induced retinopathy (OIR)

C57BL/6 J (WT) mice (Kyudo Company, Tosu, Saga, Japan) and IL-12p40 knock-out (KO) mice (Jackson Laboratory, Bar Harbor, ME, USA) were used for the animal experiments in the study. The animal experiments were handled according to the guidelines of Association for Research in Vision and Ophthalmology (ARVO) Statement on the Use of Animals in Ophthalmic and Vision Research. The experimental procedures were approved by the Institutional Animal Care and Use Committee of Kyushu University.

OIR was induced in mice as described in detail^2^,^53^,^54^. Briefly, postnatal day 7 (P7) pups were exposed to 75% oxygen for 5 days, and then returned to room air at P12. After the mice were sacrificed by cervical dislocation, the eyes were enucleated and used for the following experiments.

### Quantitative Real-time RT-PCR

Total RNA was extracted from the homogenized retinas or the cells by a MagDEA RNA kit (Precision System Science, Pleasanton, CA, USA)^55^. After quantifying the concentrations of the RNAs, complementary DNAs were synthesized by a First Strand cDNA Synthesis Kit (Roche, Mannheim, Germany). Quantitative real-time RT-PCR was performed and analyzed by FastStart Essential DNA Probes Master (Roche) and Taqman^®^ gene expression assays (Applied Biosystems, Foster City, CA, USA) in a LightCycler^®^ 96 Real-time PCR System (Roche) as described^3^. GAPDH was used as the endogenous control. The reference numbers for the assays were: Mm99999915_g1 (GAPDH), Mm00434165_m1 (IL-12α), Mm00434174_m1 (IL-12β), Mm01168134_m1 (IFN-γ), Mm00445235_m1 (CXCL10), Mm00434946_m1 (CXCL9), Mm01281449_m1 (VEGFA), Mm00433287_m1 (bFGF), Hs02758991_g1 (GAPDH), Hs00900055_m1 (VEGFA), Hs00266645_m1 (FGF2), and Hs00989291_m1 (IFN-γ).

### Immunofluorescent Staining

Retinas were isolated from the eyecups as described^3^, which used for flat-mounts. For other eyes, sections (20 μm) were cut using a cryostat (Leica CM 1800, Bannockburn, IL, USA). Phosphate buffered saline and Tween (PBST) was used for rinsing. After blocking by 1% BSA, the samples were incubated with the primary antibodies or conjugated antibodies overnight at 4 °C. Some samples were incubated with the second antibodies on the next day. Finally, the flat-mounted retinas and cryostat sections were covered with aqueous mounting medium (Thermo Scientific, Fremont, CA, USA).

The flat-mounts were incubated with fluorescein-labeled isolectin B4 (1:150 dilution; Vector Laboratories, Burlingame, CA). Then, we measured the sizes of the neovascular tufts and avascular areas according to a reported protocol^2^,^54^. The following antibodies were used for incubating cryostat sections: Alexa Fluor^®^ 647 anti-mouse CD4 (1:50 dilution, Biolegend, SD, USA), Alexa Fluor^®^ 647 anti-mouse CD8a (1:50 dilution, Biolegend), CD31 antibody (1:20 dilution, R&D System, MN, USA) and Alexa Fluor^®^ 488 chicken anti-goat IgG (1:100 dilution, Molecular Probes, Eugene, OR, USA). Hoechst 33342 (1:1000 dilution, Molecular Probes) was used to counterstain the nuclei. The retinas or cryostat sections were photographed by a fluorescent microscope (BZ-9000; KEYENCE, Osaka, Japan) and merged using the image-joint software BZ-Analyzer (KEYENCE)^55^,^56^,^57^.

### Intravitreal injections of Recombinant IL-12 and Antibody against IFN-γ

Recombinant mouse IL-12 (rIL-12) was diluted in PBS to 0, 5, 20, and 100 ng/μL (R&D Systems) and then injected intravitreally on P12 (0.5 μL/eye). In another experiment, anti-IFN-γ neutralizing antibody (R&D Systems) was used. Briefly, rIL-12 (5 ng/μL) was mixed with anti-IFN-γ antibody (0.5 μg/μL) in the same proportion, and then injected intravitreally on P12 (1 μL/eye). Non-immune IgG (R&D Systems) was used as the control. A 33-gauge needle on a Hamilton syringe was used for the intravitreal injections. Eyes were enucleated and analyzed at P17.

### Culture of HRECs

Human retinal microvascular endothelial cells (HRECs; Cell Systems Corporation, Kirkland, WA, USA) were maintained with CS-C complete medium (Cell Systems Corporation) in 6% CO_2_ at 37 °C. Cells at passages 5–7 were used for the studies.

HRECs suspensions (2 × 10^4^ cells/500 μL) were seeded in 24 wells plates, cultured in C-SC medium with 10% FBS, and allowed the attachment. After starvation in C-SC medium with 1% FBS for 24 h, the medium was replaced with or without recombinant human IL-12 (rIL-12) at 0.1 ng, 1 ng, or 10 ng/mL (R&D System) for 24 h. Then the cells were collected and examined for the mRNA expressions by real-time RT-PCR.

### Tube Formation Assays for HRECs

Tube formation studies were performed as described in detail^3^,^58^,^59^,^60^. Briefly, growth factor-reduced Matrigel matrix (BD Biosciences, Franklin Lakes, NJ, USA) was aliquoted into each well of a 96-wells plate for 30 min at 37 °C. After gel formation has occurred, serum starved HRECs were suspended in C-SC medium, and seeded at a final density of 2 × 10^4^ cells/100 μL. The cells were cultured with or without recombinant human IL-12 (rIL-12) at 0.1 ng, 1 ng, and 10 ng/mL (R&D System). In another experiment, recombinant human MIG/CXCL9 (100 ng/mL, R&D System) and IP-10/CXCL10 (100 ng/mL, R&D System) were added to the medium of the cells. We examined six independent wells for each group. After culturing for 24 h, two representative images per well were photographed randomly by a digital camera with a phase contrast microscope (Olympus CK2, Olympus, Tokyo, Japan). Angiogenesis Analyzer for NIH ImageJ software (http://rsb.info.nih.gov/ij) was used for the final analyses.

### Statistical Analyses

In the present study, the results are presented as the means ± standard error (SEMs). The statistical analyses of the differences between each group were determined by Student’s *t* tests or one-way ANOVA that followed by Dunnett’s tests. *P* < 0.05 was considered statistically significant. Statistical analyses were performed using JMP 10.0.2 (SAS Institute, Cary, NC, USA).

## Additional Information

**How to cite this article**: Zhou, Y. *et al.* Interleukin-12 inhibits pathological neovascularization in mouse model of oxygen-induced retinopathy. *Sci. Rep.* **6**, 28140; doi: 10.1038/srep28140 (2016).

## Supplementary Material

Supplementary Information
