# Supplementary material for: Interleukin-12 inhibits pathological neovascularization in mouse model of oxygen-induced retinopathy
Source: Sci Rep. 2016 Jun 17;6:28140. doi: 10.1038/srep28140 (PMC4911585; doi:10.1038/srep28140)
Supplement: Supplementary Information [file srep28140-s1.doc]

SUPPLEMENTARY INFORMATION

**Interleukin-12 inhibits pathological neovascularization in mouse model of oxygen-induced retinopathy**

Yedi Zhou, Shigeo Yoshida, Yuki Kubo, Yoshiyuki Kobayashi, Takahito Nakama, Muneo Yamaguchi, Keijiro Ishikawa, Shintaro Nakao, Yasuhiro Ikeda, Tatsuro Ishibashi, Koh-Hei Sonoda


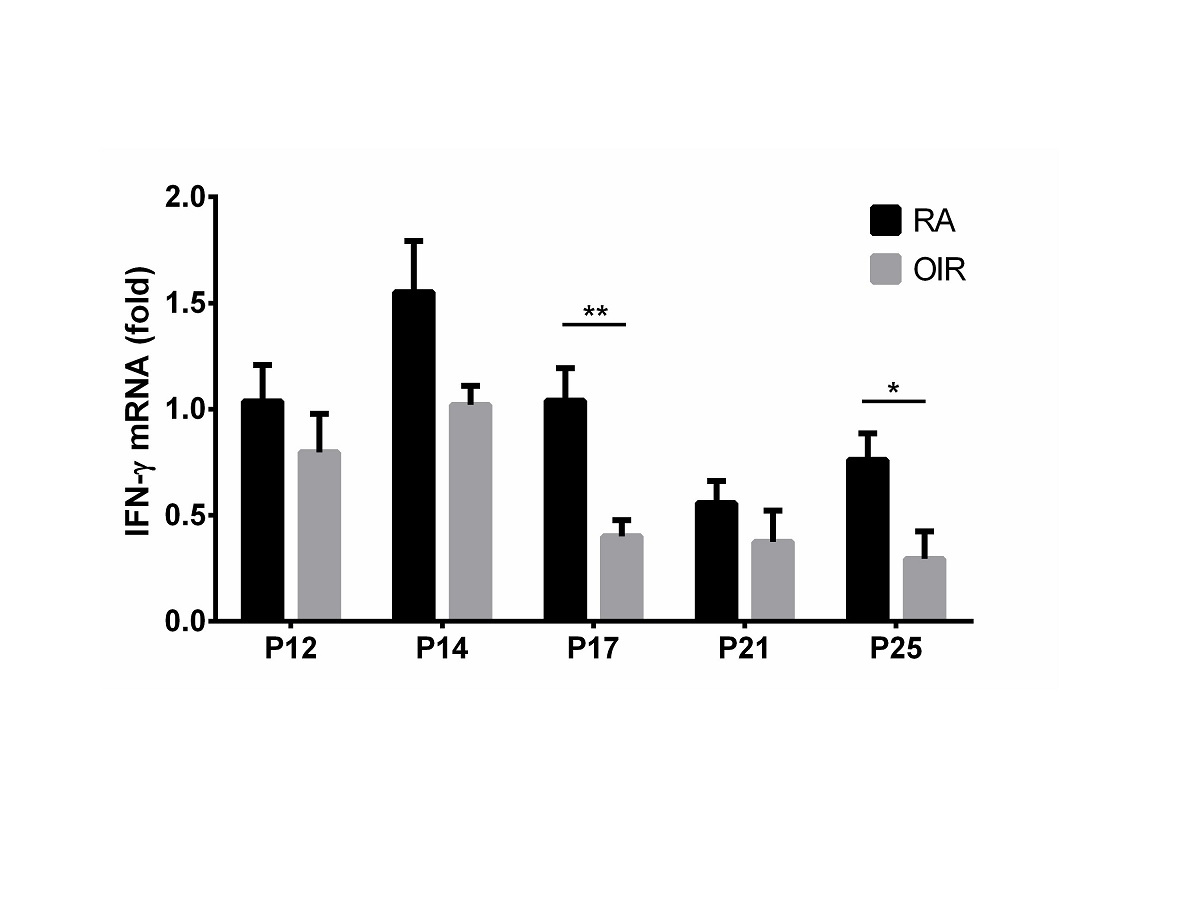


**Supplementary Figure S1.** Expression of the mRNAs of IFN-γ determined by real-time RT-PCR in mice with oxygen-induced retinopathy (OIR).

Expression of IFN-γ was significantly decreased at P17 and P25.

***P* <0.01, **P* <0.05 compared to the room air controls at the same time points (n = 4/group).


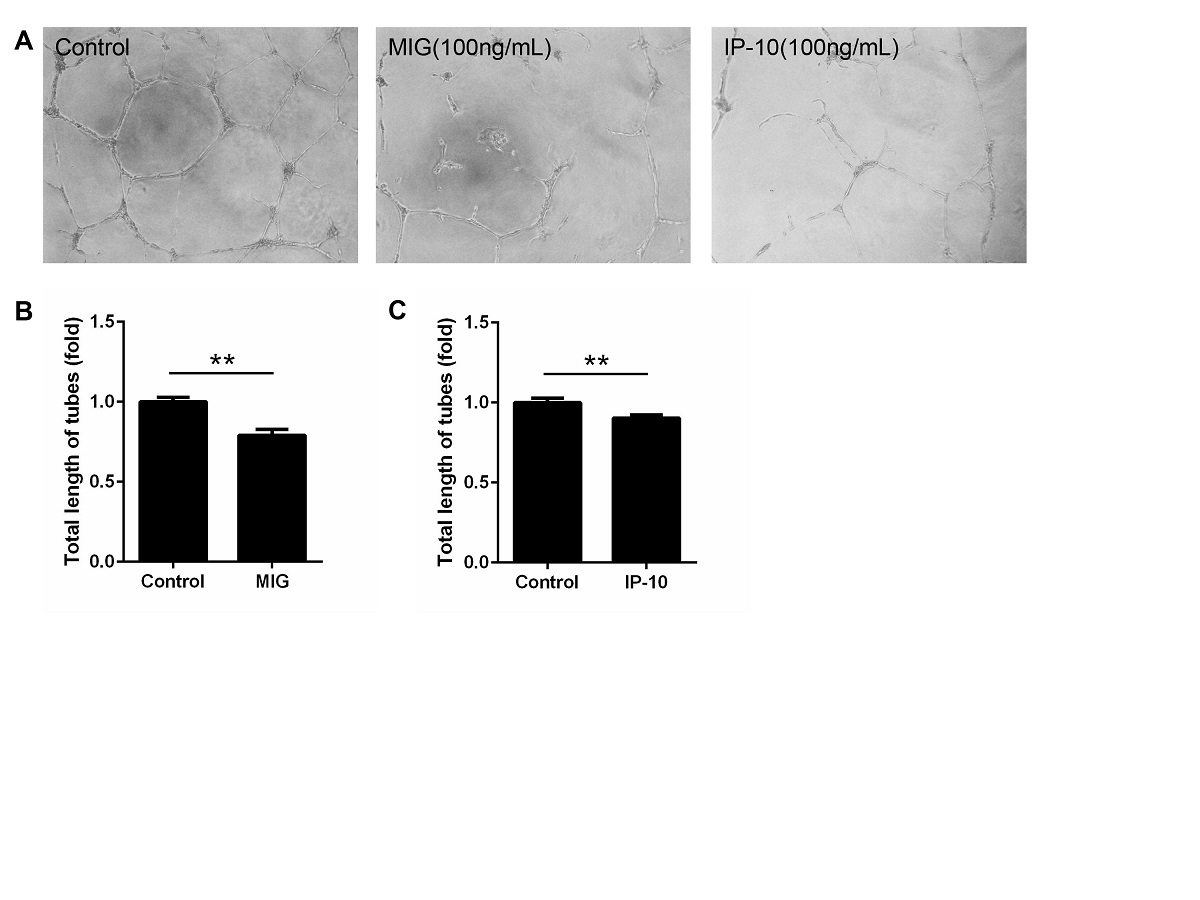


**Supplementary Figure S2.** MIG and IP-10 inhibit tube formation assay of HRECs. Photographs of tube formation were taken after cultured alone or stimulated by recombinant MIG/CXCL9 and IP-10/CXCL10 (A). The lengths of the tubes were quantitative assessed in each group. The total lengths were significantly inhibited by both MIG (B) and IP-10 (C). ***P* <0.01 compared to the control (n = 12/group).
